# Factors influencing professional life satisfaction among neurologists

**DOI:** 10.1186/s12913-017-2343-8

**Published:** 2017-06-19

**Authors:** Stephanie M. Teixeira-Poit, Michael T. Halpern, Heather L. Kane, Michael Keating, Murrey Olmsted

**Affiliations:** 1 RTI International, Research Triangle Park, Durham, NC USA; 2 RTI International, Washington, DC USA; 3 Health Services Administration and Policy, Temple University College of Public Health, Philadelphia, PA USA

**Keywords:** Personal satisfaction, Work satisfaction, Neurology, Workforce, Survey, Attitude of health personnel, Burnout, professional, Physicians/psychology

## Abstract

**Background:**

Predicted shortages in the supply of neurologists may limit patients’ access to and quality of care for neurological disorders. Retaining neurologists already in practice provides one opportunity to support the overall supply of practicing neurologists. Understanding factors associated with professional life satisfaction (and dissatisfaction) and implementing policies to enhance satisfaction may encourage neurologists to remain in clinical practice. In this paper, we present results from the first study examining factors associated with professional life satisfaction among a large sample of U.S, neurologists.

**Methods:**

We collaborated with the AAN to survey a sample of U.S. neurologists about their professional life satisfaction. Analyses examined the association of physician and practice characteristics with aspects of professional life satisfaction, including satisfaction with their career in medicine, medical specialty, current position, relationship with colleagues, relationship with patients, work/life balance, and pay.

**Results:**

The study population consisted of 625 neurologists. In multivariate regression analyses, no single group or population stratum indicated high (or low) responses to all aspects of satisfaction. Older neurologists reported higher satisfaction with career, specialty, and relationship with patients than younger neurologists. Female neurologists had significantly lower satisfaction with pay than male neurologists. Neurologists who spent more time in research and teaching had greater satisfaction with specialty, relationship with colleagues, and relationship with patients than those spending no time in research. Neurologists who practiced in small cities/rural areas reported lower satisfaction across multiple dimensions than those practicing in large urban areas. Neurologists in solo practice had greater satisfaction with the relationship with their patients, but lower satisfaction with pay.

**Conclusions:**

Satisfaction is a multidimensional construct that is associated with physician and practice characteristics. Enhancing professional life satisfaction among neurologists requires multiple strategies, such as promoting comparable wages for men and women, providing collaboration and research opportunities, and providing resources for small and rural practices.

**Electronic supplementary material:**

The online version of this article (doi:10.1186/s12913-017-2343-8) contains supplementary material, which is available to authorized users.

## Background

Dall and colleagues [1] predicted significant shortages in the supply of neurologists, which may limit patients’ access to and quality of care. Although recruiting medical students to pursue training in neurology provides one opportunity for increasing the supply of practicing neurologists [2], retaining those already in practice reduces the number exiting the field, thus supporting overall supply. Retaining neurologists depends, in part, on their professional life satisfaction; those satisfied with their career may opt to stay in practice while those dissatisfied may retire or reduce the number of clinic hours [3–5].

Previous research found that demographic characteristics, such as age and gender, were correlated with professional life satisfaction. Pathman et al. [4] reported that older generalist and specialist physicians reported higher satisfaction than younger physicians for 8 of 10 studied aspects. Similarly, DeMello & Deshpande [6] reported higher satisfaction among older psychiatrists compared to younger ones. However, Leigh et al. [7] reported a nonlinear relationship between physician age and satisfaction; the youngest and oldest physicians had the highest satisfaction. Unlike these findings for age, several studies found no difference in overall job satisfaction between male and female physicians [7–11]. However, in a nationally representative survey of physicians in primary and specialty nonsurgical care, McMurray et al. [12] reported that female physicians indicated significantly lower satisfaction with certain components of job satisfaction – such as pay, resources, and autonomy – than did male physicians; in addition, female physicians earned less than did male physicians. This gender-based wage difference has also been reported in other specialties, such as colorectal surgery [13].

Past studies also found that practice characteristics – such as practice arrangement, practice location, and participation in research – were predictors of professional life satisfaction. For practice arrangement, Nixon and Jaramillo [14] reported that physicians who work for HMOs and hospitals have less autonomy and decision-making power, but more satisfaction with job resources, regulatory climate, and professions than do self-employed physicians in solo and group practices. In addition, Sturm [15] reported that, among surgeons, working in small practices was the strongest predictor of career dissatisfaction; 26% of surgeons in small practices indicated dissatisfaction with their careers, double that of surgeons in other practice settings. In terms of practice location, previous research found that rural physicians who felt overworked and professionally isolated reported lower satisfaction, especially with their incomes [16]. Among rural family physicians, decreased job satisfaction was correlated with increased numbers of work hours, including hours on call, and the absence of a local hospital [17]. Participation in research was also a significant predictor of job satisfaction. Mohr and Burgess [3] found that physicians who spent time in research reported higher job satisfaction and favorable job characteristics ratings. In a survey of obstetrician-gynecologists, research and teaching were two main factors contributing to job satisfaction among academic physicians compared with private practice physicians [18].

Professional life satisfaction is a multidimensional concept that may include multiple facets of a neurologist’s work, such as medical specialty, relationships with colleagues and patients, work/life balance, and pay. Pathman et al. [4] found that addressing specific aspects of professional dissatisfaction (e.g., pay, community relationships) mattered more in retaining physicians than maintaining satisfaction generally. Thus, understanding and addressing factors associated with specific aspects of professional life satisfaction (and dissatisfaction) may encourage existing neurologists to remain in clinical practice. In this paper, we examine the factors associated with aspects of professional life satisfaction among neurologists.

## Methods

### Instrument development

We initially developed the MS Physician Workforce Neurologist Survey to assess factors associated with provision of care for multiple sclerosis (MS) patients among neurologists, in a study funded by the National Multiple Sclerosis Society. Results from these analyses have been presented previously [19]. The survey also collected information on professional satisfaction among neurologists responding to this survey. We developed the survey questions by adapting questions on professional life satisfaction that were available in existing physician career surveys, such as: (1) the Survey of Clinical Oncology Fellows Completing Training [20]; (2) the New York State Education Department Physician Survey [21]; (3) Outlook for the Future of the Obstetrician Gynecologist Workforce [9]; and (4) the Physician Worklife Survey [22]. We selected and adapted existing questions and crafted new items to create a draft survey, which included questions on respondents’ professional life satisfaction, demographic characteristics, and practice characteristics.

To ensure comprehensiveness and usability of the survey, we sought input from an advisory panel of experts identified for this study and from the American Academy of Neurology (AAN) MS Section Executive Committee. The advisory panel, including neurologists, reviewed and provided comments on the survey, which was revised based on their feedback. Members of the AAN MS Section Executive Committee then reviewed the survey to ensure that it was feasible for AAN members to complete.

The survey was reviewed by the RTI International Institutional Review Board prior to distribution and determined to be low risk to participants. A statement at the beginning of the survey indicated that participants were being asked to respond to the survey as part of a research study on factors related to the workforce of medical providers for multiple sclerosis. The statement indicated that participation was voluntary and that, by responding to the survey, they were consenting to participate in the research. This form of consent (as opposed to requiring a signed consent form) was permissible because the survey collected only attitudes, opinions, and a few characteristics (demographic and practice) from participants. All participants were adults over the age of [18]. The full survey is available in Additional file 1.

### Study population and survey administration

The sampling frame consisted of neurologists practicing in the United States who were members of the AAN. We collaborated with the AAN, which administered the survey and collected responses. The AAN contacted a random sample of 1700 neurologists who resided in the United States and were members of the AAN, excluding retired members; those in medical school, residency, or fellowship; and AAN committee members who participated in survey review.

The AAN administered the survey via e-mail, postal mail, or fax to the neurologist sample in January 2012. The AAN sent neurologists an e-mail describing the study and including a hyperlink to the online version of the survey. The paper version included a cover letter signed by the Chair of the AAN MS Section. AAN staff tracked responses to determine whether invited neurologists submitted surveys and, for nonresponders, distributed two e-mail reminders to participate and two faxed or mailed paper versions of the survey. AAN assigned a personal identification number to each respondent and, therefore, duplicate responses were not permitted. Of the 1693 neurologists invited to participate, 662 submitted responses (response rate of 39.1%).

### Secondary data

We used masked identification numbers (assigned by the AAN) to merge de-identified neurologists’ survey responses with demographic and practice characteristics from the *2008 AAN Member Census*. From the *Census*, practice arrangements were categorized as solo practice, neurology group, multispecialty group, university-based group, other, and unknown. The “other” category included staff-model HMO, government hospital/clinic, and other public or private hospital or clinic setting.

### Dependent variables

The dependent variables for this analysis were self-reported aspects of professional life satisfaction. The survey captured information on seven aspects of respondent’s professional life satisfaction: career in medicine, medical specialty, current position, relationship with colleagues, relationship with patients, personal time off (work/life balance), and pay. Respondents scored their satisfaction with aspects of their professional life on a five-point Likert-scale ranging from “not at all satisfied” to “extremely satisfied”. The question wording for each measure is available Additional file 1, which provides the full survey.

### Independent variables

Independent variables included demographic and practice characteristics. Self-reported demographic characteristics included age (categorized in quartiles), sex, race (White, Asian, and Other Race with Black/African American, American Indian/Alaska Native, and Native Hawaiian/Other Pacific Islander groups combined because of small numbers), and ethnicity (Hispanic/Latino or not). Survey respondents were categorized as completing any neurology subspecialty training if they responded “Yes, I completed a neurology subspecialty fellowship” or “Yes, I participated in informal mentoring or training in a subspecialty area of neurology but did not complete a formal fellowship” to the question “Have you completed any neurology subspecialty training?”

Self-reported practice characteristics included weeks per year providing direct patient care (categorized in quartiles), percent time devoted to research/teaching (categorized in tertiles), percent time devoted to administration/other (categorized in tertiles), total number of patients seen in an average week (categorized in quartiles), total number of new patients seen in an average week (categorized in quartiles), and plans to retire from patient care or significantly reduce patient care hours (categorized as yes or no). Percent time devoted to clinical practice was coded into three categories: less than 50, 50 to 75%, and more than 75% of my professional time. Urban/rural designation of practice area was coded into three categories: within a major city, suburban or moderate-sized city, and small city or rural area.

### Analyses

We computed bivariate analyses to examine factors associated with each aspect of professional life satisfaction. Because the dependent variables were not normally distributed (Shapiro-Wilk W tests for normality, *p* < 0.001), we computed nonparametric bivariate analyses to examine factors associated with satisfaction using Wilcoxon-Mann-Whitney tests for independent variables with two categories and Kruskal Wallis tests for independent variables with three or more categories. We performed ordered logistic regressions to identify predictors of greater scores for aspects of professional life satisfaction while controlling for physician and practice characteristics. We selected ordered logistic regression because it is an appropriate statistical technique to use for regression analyses of ordered, categorical dependent variables [23–26], and is particularly well suited to analysis of dependent variables with a small number of categories [27]. Ordered logistic regression models make full use of the information present in an ordinal scale [26]. Although ordinary least square (OLS) models have also been used for analyses of ordinal dependent variables, this approach can be problematic. OLS models applied to ordinal dependent variables may produce unbiased parameter estimates, but the corresponding estimates of variance may be biased and inconsistent. This may lead to underestimating the impacts of certain independent variables [25, 26], further supporting the use of ordinal logistic regression for ordered dependent variables.

In our analyses, the ordered logistic regression models included all independent variables that were significantly (*p* < 0.05) or marginally significantly (0.05 < *p* < 0.10) associated with any aspect of professional life satisfaction in bivariate analyses (see Additional file 2: Table S1). All variables included in regression analyses were examined for multicollinearity (VIF > 5) prior to inclusion in the final model. Plans to retire from patient care or significantly reduce patient care hours was excluded from multivariate models for collinearity with respondent’s age. Missing data generally comprised less than 9% of responses; individuals with missing data were excluded from analyses.

## Results

### Population characteristics

The study population consisted of 625 neurologists (Table 1). The mean age of neurologists was 52 years. About 70% of neurologists were males, and 78% identified as white. The majority of neurologists were primarily engaged in clinical practice (two-thirds indicated spending more than 75% of their time in clinical practice). Only 4% of respondents reported that they did not have a clinical practice. Of the remaining neurologists, 28 % considered a neurology group to be their primary practice arrangement compared to 26% in solo practice, 17% in a university based group, 12% in a multispecialty group, and 12% in some other practice arrangement. Half of neurologists practiced within a major city, and one-third practiced in a suburban or moderate-sized city. The study population has similar demographic characteristics to the population of neurologists who are members of the American Academy of Neurology (AAN), which has a mean age of 53 years, 76% males, 80% who identify as white, 28% in a neurology group, 24% in solo practice, and 36% in multispecialty/university settings [28]. Neurologists in the study population indicated the highest satisfaction with their relationship with patients (mean = 4.20 out of a 5-point Likert-scale) and lowest satisfaction with their pay (mean = 2.97 out of a 5-point Likert-scale; Table 2).

**Table 1:** Characteristics of the study population^b^

Study Population Characteristic	n	%
Total study population	625	100.00
Age		
44 years or younger	160	26.02
45 to 53 years	160	26.02
54 to 59 years	153	24.88
60 years or older	142	23.09
Sex		
Male	439	70.47
Female	184	29.53
Race		
White	404	78.45
Asian	102	19.81
Other race	15	2.91
Ethnicity		
Hispanic or Latino	34	6.76
Not Hispanic or Latino	469	93.24
Completed any neurology subspecialty training		
Yes	467	76.18
No	146	23.82
Faculty member in a medical school, neurology department, or other academic department		
Yes	175	67.41
No	362	32.59
Weeks per year providing direct patient care		
0 to 45	130	21.17
46 to 48	222	36.16
49 to 50	170	27.69
51 to 52	92	14.98
Primary practice arrangement		
Solo practice	140	26.32
Neurology group	151	28.38
Multispecialty group	62	11.65
University based group	94	17.67
Other^a^	64	12.03
I do not have a clinical practice	21	3.95
% of time devoted to clinical practice		
Less than 50% of my professional time	69	12.80
50% to 75% of my professional time	107	19.85
More than 75% of my professional time	363	67.35
% of time devoted to research/teaching		
None of my professional time	204	37.85
Under 25% of my professional time	222	41.19
25% or more of my professional time	113	20.96
% of time devoted to administration/other		
None of my professional time	244	45.27
Less than 10% of my professional time	148	27.46
10% or more of my professional time	147	27.27
Practice location		
Within a major city	316	51.38
Suburban or moderate-sized city	221	35.93
Small city/rural area	78	12.68
Total number of patients seen in an average week		
0 to 34	146	24.70
35 to 50	156	26.40
51 to 75	154	26.06
76 to 400	135	22.84
Total number of new patients seen in an average week		
0 to 8	155	26.45
9 to 14	146	24.91
15 to 20	171	29.18
21 to 120	114	19.45
Plans to retire from patient care or significantly reduce patient care hours		
Yes	62	10.08
No	553	89.92

^a^The “other” category includes neurologists who responded “staff-model HMO,” “government hospital or clinic,” or “other public or private hospital or clinic setting” to the question, “Indicate in which practice arrangement you spend the majority of your clinical time.”

^b^Values may not sum to the full study population due to missing responses

**Table 2:** Satisfaction among neurologists with their professional life

Aspects of Professional Life Satisfaction	n	Mean	Standard Deviation
Your career in medicine	621	3.78	0.97
Your medical specialty	623	4.07	0.92
Your current position	618	3.75	0.99
Relationship with colleagues	622	3.94	0.86
Relationship with patients	619	4.20	0.75
Personal time off (work/life balance)	616	3.11	1.28
Pay	613	2.95	1.17

### Regression analyses of characteristics associated with aspects of professional life satisfaction

As discussed in the Methods section, we used ordered logistic regression models to identify physician and practice characteristics significantly associated with each aspect of professional life satisfaction while controlling for the other characteristics listed in Table 3. Neurologists who were 60 years or older were significantly more likely to have higher satisfaction with their career, specialty, and relationship with patients compared with those 44 years or younger. Neurologists 54 to 59 years were significantly less likely to be satisfied with their pay. Female neurologists were significantly more likely to have higher satisfaction with their relationship with patients compared with males.

**Table 3:** Odds ratios for ordered logistic regressions predicting greater professional life satisfaction

Study Population Characteristic	Aspects of Professional Life Satisfaction						
	Your career in medicine	Your medical specialty	Your current position	Relationships with colleagues	Relationships with patients	Personal time off (work/life balance)	Pay
Age (Reference: “44 years or younger”)							
45 to 53 years	1.06	0.82	0.91	0.81	1.09	0.77	0.80
54 to 59 years	1.18	1.00	0.86	0.93	1.20	0.66	0.53†
60 years or older	1.81†	2.30†	1.17	1.15	2.56†	1.39	1.12
Sex (Reference: “Male”)							
Female	0.98	1.26	0.79	1.11	1.80†	0.90	0.70
Race							
(Reference: “Non-White”): White	1.14	0.74	0.48	0.34	0.79	1.72	0.88
(Reference: “Non-Asian”): Asian	2.43	0.93	0.78	0.58	0.85	3.28	1.57
(Reference: “Non-other race”): Other race	1.22	1.01	0.39	0.34	0.84	1.67	0.45
Faculty member in a medical school, neurology department, or other academic department (Reference: “No”)							
Yes	1.47	1.22	1.29	1.31	1.51	1.40	0.88
Weeks per year providing direct patient care (Reference: “0 to 45”)							
46 to 48	0.89	0.87	1.16	1.72†	0.88	0.98	0.80
49 to 50	0.77	0.86	1.09	1.15	0.93	0.79	1.28
51 to 52	1.02	1.14	1.18	1.38	1.11	0.74	0.86
Primary practice arrangement (Reference: “Solo practice”)							
Neurology group	0.75	0.61†	0.92	1.29	0.63	0.81	1.31
Multispecialty group	1.28	1.47	1.56	1.67	0.45†	1.24	2.87†
University-based group	0.83	0.67	0.84	0.94	0.48	0.70	1.01
Other^a^	1.37	0.87	1.43	1.19	0.43†	1.41	3.75†
I do not have a clinical practice	0.75	1.70	0.78	0.57	0.77	1.70	3.04
% of time devoted to clinical practice (Reference: “Less than 50% of my professional time”)							
50% to 75% of my professional time	0.68	1.00	0.47	0.66	1.05	0.90	1.11
More than 75% of my professional time	0.82	1.01	0.56	0.76	1.10	1.39	0.85
% of timed to research/teaching (Reference: “None of my professional time”)							
Under 25% of my professional time	1.20	1.45	1.21	1.89†	1.55	0.85	1.08
25% or more of my professional time	1.54	2.52†	1.18	2.06	2.45†	1.19	0.88
Practice location (Reference: “Within a major city”)							
Suburban or moderate-sized city	0.88	0.95	0.84	1.14	1.40	1.01	0.70
Small city/rural area	0.57	0.54†	0.60	0.77	0.61	0.55†	0.83
Total number of patients seen in an average week (Reference: “0 to 34”)							
35 to 50	0.96	0.83	1.10	1.16	1.24	0.86	0.79
51 to 75	1.33	1.32	1.31	1.13	1.75	0.67	1.03
76 to 400	1.08	1.12	1.46	0.73	1.09	0.67	0.87

† Statistically significant, *p* < 0.05

^a^The “other” category includes neurologists who responded “staff-model HMO,” “government hospital or clinic,” or “other public or private hospital or clinic setting” to the question, “Indicate in which practice arrangement you spend the majority of your clinical time.”

Neurologists who spent fewer weeks per year providing direct patient care were significantly more likely to have higher satisfaction with their relationship with colleagues Those practicing in neurology groups had significantly lower likelihood of satisfaction with their medical specialty, while neurologists in multispecialty groups or other settings had significantly lower likelihood of satisfaction with their relationship with patients but higher likelihood of satisfaction with their pay. Neurologists who participate in research/teaching had higher likelihood of satisfaction with their specialty, relationship with colleagues, and relationship with patients compared with those who spent none of their professional time in research/teaching. Finally, neurologists practicing in small cities/rural areas had decreased likelihood of satisfaction with their specialty and personal time off compared with neurologists practicing within major cities.

Race and faculty member status were not significantly associated with any aspect of professional life satisfaction while controlling for other factors in the regression analyses.

## Discussion

Our results indicate that professional life satisfaction among neurologists is a multifaceted metric with no single group of neurologists indicating high (or low) responses to all aspects of satisfaction. Differing groups may indicate greater likelihood of higher scores for some aspects of satisfaction, but no significant differences or even decreases likelihood for other aspects. For example, neurologists in multispecialty group practices reported significantly lower likelihood of satisfaction regarding relationship with patients compared with those in solo practice, but significantly higher likelihood of satisfaction regarding pay (Table 3). If we examined the relationship of neurologists’ demographic and practice characteristics with satisfaction using a single overall satisfaction measure, we would likely have missed these significant differences in associations and the additional information they provide.

Neurologists who were 60 years of age or older reported higher likelihood of satisfaction with career, specialty, and relationship with patients compared with those aged 44 or younger (Table 3). This may reflect increased stability or confidence that occurs with greater experience or long-term participation in medical practice compared with physicians who are newer to medical practice. Older physicians may have greater autonomy regarding their careers and future plans, increasing career satisfaction. Previous studies of physician satisfaction found correlations between age and satisfaction. Although two studies found that older clinicians reported higher satisfaction than younger clinicians [4, 6] Leigh et al. [7] reported a nonlinear relationship between physician age and satisfaction.

Female neurologists had lower average satisfaction with pay than did males (2.80 vs. 3.01, respectively, *p* < 0.05), and marginally lower likelihood (0.05 < *p* < 0.10) of satisfaction with pay compared with males in regression analyses (results not shown). While not reaching statistical significance in regression analyses, this finding may reflect wage inequality because female neurologists receive less pay than male neurologists. In 2012, compensation averaged $160,000 for female neurologists and $198,000 for males; only part of this difference was attributable to more female physicians working part-time [29] Wage inequality is not unique to neurology; a gender-based wage difference has been reported across medical care and in other specialties. Among newly trained physicians in New York, females made $16,819 less on average than males, and this wage inequality was not explained by specialty choice, practice setting, or work hours [30]. Research on specialties report similar trends with female colorectal surgeons and dermatologists earning less than males [13, 31], although the difference among dermatologists was not significant when controlling for other factors [31]. The finding of decreased satisfaction with pay among female neurologists is consistent with other studies on physician satisfaction. Addressing pay equity by improving female neurologist remuneration may be one strategy to improve satisfaction.

Neurologists who participate in research and teaching had higher likelihood of satisfaction with specialty, relationship with colleagues, and relationship with patients than those not participating in these activities (Table 3). This may reflect that neurologists who participate in research and teaching often engage in collaborative interactions and “team science” or opportunities to interact with colleagues in an intellectually stimulating environment and to explore new interventions that may improve patient outcomes. Similarly, participation in certain types of research (e.g., clinical trials) may involve more in-depth interactions with patients, which could lead to higher satisfaction with professional life. Previous research found that spending time in research contributes to higher job satisfaction among physicians [3, 18]. Research opportunities for neurology residents may stimulate research participation during subsequent careers and subsequently increase job satisfaction [32].

Neurologists who practiced in small cities/rural areas had lower likelihood of satisfaction across multiple dimensions compared with those practicing in large urban areas. Neurologists practicing in small cities or rural areas likely have fewer practice-related resources, including less funding, fewer colleagues, and fewer support services. Previous research found that rural physicians who felt overworked and professionally isolated reported lower satisfaction, especially with their incomes [16]. Among rural family physicians, decreased job satisfaction was correlated with increased numbers of work hours, including hours on call, and the absence of a local hospital [17]. Providing high-quality neurologic care in the context of physician shortages is challenging in rural areas.

Compared with neurologists in multispecialty groups and the “other” practice setting category, neurologists in solo practice (approximately 26% of the study population) had significantly higher likelihood of satisfaction regarding their relationship with patients, but significantly lower likelihood of satisfaction with pay. Those in solo practice may have the ability to spend more time with their patients, but likely have fewer resources, fewer support personnel, and lower income. There has been only limited previous work examined the impacts of practice arrangement on physician satisfaction, and (to our knowledge), no previous work examined this relationship among neurologists. However, research among surgeons suggests that those working in small practices had greater dissatisfaction with their careers compared to those working in other practice arrangements [15].

This study has several limitations. Although the neurologists invited to participate in the survey were randomly selected from the relevant members of the AAN, only 39% responded to the survey; this group may not be representative of U.S. neurologists in general. All information provided by survey participants was by self-report; we did not attempt to validate any responses. In addition, as with all surveys, we limited the number of items asked to minimize respondent burden. There are likely additional factors that influence the professional life satisfaction of neurologists that were not captured in our survey.

## Conclusions

This study provides important information on factors influencing satisfaction among neurologists. Satisfaction is a multidimensional construct that is significantly associated with demographic and practice characteristics; however, specific neurologist or neurology practices characteristics are not consistently associated with all aspects of satisfaction. Our findings suggest that enhancing professional life satisfaction among neurologists (and potentially retaining them in the field) will require multiple strategies, such as providing increased collaboration and research opportunities, enhancing resources available for small and rural practices, improving female neurologist remuneration to make it equal to that of male neurologists, and exploring alternate funding policies for practices in rural and underserved areas.

This study suggests several avenues for future research. Given the existing and projected future shortages among neurologists, research should explore which strategies optimize satisfaction among neurologists and how this affects physician recruitment and retention. Research is also needed to identify strategies for improving satisfaction among rural neurologists or identifying alternative approaches (e.g., telemedicine) for providing neurologic care in these areas. Future research exploring alternative funding policies for neurologists in solo practice, particularly those in rural and other underserved areas, may be useful for identifying strategies to improve satisfaction.

## Additional files

Additional file 1: MS Physician Workforce Neurologist Survey. Additional file 1 contains the MS Physician Workforce Neurologist Survey that the authors used to survey a sample of U.S. neurologists about their professional life satisfaction. (DOCX 37 kb)

Additional file 2: Table S1. Mean Professional Life Satisfaction by Study Population Characteristics. **Table S1.** contains bivariate analyses displaying the association of independent variables with aspects of professional life satisfaction. Independent variables that were significantly (*p* < 0.05) or marginally significantly (0.05 < *p* < 0.10) associated with any aspect of professional life satisfaction were included in ordered logistic regression models. (DOCX 17 kb)

## Abbreviations

AAN: American Academy of Neurology
MS: Multiple sclerosis
