# Supplementary material for: Factors influencing professional life satisfaction among neurologists
Source: BMC Health Serv Res. 2017 Jun 19;17:409. doi: 10.1186/s12913-017-2343-8 (PMC5477134; doi:10.1186/s12913-017-2343-8)
Supplement: Supplementary file 2 [file 12913_2017_2343_MOESM2_ESM.docx]

Additional file 2: Table S1. Mean Professional Life Satisfaction by Study Population Characteristics

| Study Population Characteristic | Aspects of Professional Life Satisfaction |
| --- | --- |
| Your career in medicine | Your medical specialty | Your current position | Relationships with colleagues | Relationships with patients | Personal time off (work/life balance) | Pay |
| Age |
| 44 years or younger | 3.72 | 4.01† | 3.74 | 3.92 | 4.08† | 3.19† | 3.12† |
| 45 to 53 years | 3.71 | 3.95† | 3.71 | 3.90 | 4.15† | 3.00† | 2.83† |
| 54 to 59 years | 3.73 | 4.03† | 3.69 | 3.92 | 4.17† | 2.87† | 2.76† |
| 60 years or older | 3.96 | 4.33† | 3.86 | 4.03 | 4.41† | 3.34† | 3.11† |
| Sex |
| Male | 3.76 | 4.04 | 3.78 | 3.93 | 4.16† | 3.11 | 3.01† |
| Female | 3.81 | 4.11 | 3.67 | 3.96 | 4.29† | 3.09 | 2.80† |
| Race |
| White | 3.71† | 4.07 | 3.70 | 3.89 | 4.20 | 3.01† | 2.88 |
| Asian | 4.00† | 4.12 | 3.92 | 4.07 | 4.22 | 3.44† | 3.18† |
| Other race | 3.73 | 4.20 | 3.53 | 3.80 | 4.27 | 3.27 | 2.60 |
| Ethnicity |
| Hispanic or Latino | 3.82 | 4.26 | 3.85 | 3.82 | 4.29 | 2.97 | 2.78 |
| Not Hispanic or Latino | 3.76 | 4.06 | 3.72 | 3.94 | 4.20 | 3.08 | 2.93 |
| Completed any neurology subspecialty training |
| Yes | 3.76 | 4.07 | 3.73 | 3.92 | 4.19 | 3.10 | 2.95 |
| No | 3.83 | 4.06 | 3.80 | 4.03 | 4.21 | 3.13 | 2.94 |
| Faculty member in a medical school, neurology department, or other academic department |
| Yes | 3.97† | 4.23† | 3.85 | 4.11† | 4.36† | 3.25 | 2.90 |
| No | 3.68† | 4.00† | 3.70 | 3.86† | 4.14† | 3.04 | 2.94 |
| Weeks per year providing direct patient care |
| 0 to 45 | 3.95 | 4.23 | 3.79 | 3.91 | 4.20 | 3.37† | 3.13 |
| 46 to 48 | 3.75 | 4.04 | 3.74 | 4.05 | 4.19 | 3.15† | 2.94 |
| 49 to 50 | 3.67 | 3.98 | 3.73 | 3.83 | 4.18 | 2.91† | 2.95 |
| 51 to 52 | 3.80 | 4.11 | 3.78 | 3.94 | 4.24 | 3.00† | 2.78 |
| Primary practice arrangement |
| Solo practice | 3.71† | 4.06† | 3.66 | 3.84 | 4.34 | 3.00 | 2.64† |
| Neurology group | 3.56† | 3.83† | 3.66 | 3.90 | 4.14 | 2.90 | 2.83† |
| Multispecialty group | 3.94† | 4.24† | 3.98 | 4.07 | 4.10 | 3.26 | 3.35† |
| University based group | 3.95† | 4.22† | 3.83 | 4.09 | 4.30 | 3.23 | 2.85† |
| Other* | 4.06† | 4.23† | 3.87 | 4.05 | 4.17 | 3.41 | 3.37† |
| I do not have a clinical practice | 3.71† | 4.24† | 3.52 | 3.62 | 3.85 | 3.33 | 3.30† |
| % of time devoted to clinical practice |
| Less than 50% of my professional time | 3.97 | 4.28† | 3.84 | 4.01 | 4.18 | 3.30 | 3.00 |
| 50% to 75% of my professional time | 3.84 | 4.19† | 3.70 | 4.00 | 4.31 | 3.09 | 3.02 |
| More than 75% of my professional time | 3.71 | 4.01† | 3.74 | 3.91 | 4.18 | 3.07 | 2.88 |
| % of time devoted to research/teaching |
| None of my professional time | 3.61† | 3.91† | 3.65 | 3.76† | 4.09† | 3.09 | 2.86 |
| Under 25% of my professional time | 3.82† | 4.13† | 3.80 | 4.06† | 4.27† | 3.02 | 2.96 |
| 25% or more of my professional time | 3.96† | 4.27† | 3.80 | 4.03† | 4.29† | 3.28 | 2.97 |
| % of time devoted to administration/other |
| None of my professional time | 3.75 | 4.04 | 3.75 | 3.97 | 4.17 | 3.07 | 2.89 |
| Less than 10% of my professional time | 3.83 | 4.20 | 3.78 | 3.92 | 4.27 | 3.22 | 2.87 |
| 10% or more of my professional time | 3.74 | 4.02 | 3.70 | 3.92 | 4.21 | 3.03 | 3.03 |
| Practice location |
| Within a major city | 3.88† | 4.14† | 3.81† | 4.00 | 4.21 | 3.17† | 3.00 |
| Suburban or moderate-sized city | 3.75† | 4.05† | 3.75† | 3.94 | 4.27 | 3.14† | 2.92 |
| Small city/rural area | 3.42† | 3.81† | 3.50† | 3.73 | 3.99 | 2.73† | 2.82 |
| Total number of patients seen in an average week |
| 0 to 34 | 3.86 | 4.18 | 3.75 | 4.03 | 4.17 | 3.33† | 3.04 |
| 35 to 50 | 3.73 | 3.96 | 3.71 | 3.97 | 4.26 | 3.18† | 2.92 |
| 51 to 75 | 3.82 | 4.06 | 3.75 | 3.95 | 4.26 | 3.01† | 3.04 |
| 76 to 400 | 3.65 | 4.03 | 3.78 | 3.78 | 4.10 | 2.88† | 2.83 |
| Total number of new patients seen in an average week |
| 0 to 8 | 3.83 | 4.15 | 3.75 | 3.97 | 4.26 | 3.16 | 2.91 |
| 9 to 14 | 3.69 | 4.00 | 3.68 | 3.91 | 4.23 | 3.25 | 2.99 |
| 15 to 20 | 3.73 | 3.99 | 3.71 | 3.89 | 4.13 | 3.00 | 2.90 |
| 21 to 120 | 3.82 | 4.10 | 3.84 | 3.97 | 4.19 | 2.96 | 3.06 |
| Plans to retire from patient care or significantly reduce patient care hours |
| Yes | 3.52† | 3.95 | 3.44† | 3.93 | 4.27 | 2.59† | 2.75 |
| No | 3.81† | 4.08 | 3.79† | 3.94 | 4.19 | 3.16† | 2.98 |

Note: Wilcoxon-Mann-Whitney tests were calculated for independent variables with two categories. Kruskal Wallis tests were calculated for independent variables with three or more categories. 

†	Statistically significant, p < 0.05

*	The “other” category includes neurologists who responded “staff-model HMO,” “government hospital or clinic,” or “other public or private hospital or clinic setting” to the question, “Indicate in which practice arrangement you spend the majority of your clinical time.”
